# Epidemiology and treatment of malignant ovarian germ cell and sex cord stromal tumors in germany: a population-based cancer registry study from 2016 – 2021

**DOI:** 10.1038/s41598-025-32998-5

**Published:** 2025-12-18

**Authors:** Dennis Jung, Sinaida Branscheidt, Ian Wittenberg, Andrea Schmidt-Pokrzywniak, Daniela Piontek, Tonia Brand, Mike Klora, Sylke Ruth Zeissig, Nikola Beck, Anita Feller, Silke Hermann, Josephine Kanbach, Sabine Luttmann, Ron Pritzkuleit, Bettina Braun, Cynthia Erb, Henrik Kusche, Gabriele Robers, Kerstin Weitmann, Khalid Abnaof, Soo-Zin Kim-Wanner, Daniela Reil, Anne von Rüsten, Florian Oesterling, Andreas Stang, Cécile Ronckers, Claudia Bremensdorfer, Christina Justenhoven, Annette Hasenburg

**Affiliations:** 1 Department of Obstetrics and Gynecology, University Medical Center of Johannes Gutenberg University Mainz, Mainz, Germany; 2 Cancer Registry of Rhineland-Palatinate in the Institute for Digital Health Data gGmbH, Mainz, Germany; 3 Clinical Cancer Registry of Saxony-Anhalt, Halle (Saale), Germany; 4 Cancer Registry of Saxony, Dresden, Germany; 5 Clinical Cancer Registry of Lower Saxony, Hannover, Germany; 6 Regional Centre Würzburg, Bavarian Cancer Registry, Bavarian Health and Food Safety Authority, Würzburg, Germany; 7 Institute of Clinical Epidemiology and Biometry (ICE-B), University of Würzburg, Würzburg, Germany; 8 Epidemiological Cancer Registry Baden-Württemberg, German Cancer Research Center, Heidelberg, Germany; 9 Bremen Cancer Registry, Leibniz Institute for Prevention Research and Epidemiology - BIPS, Bremen, Germany; 10 Cancer Registry of Schleswig-Holstein, Institute of Cancer Epidemiology, University of Lübeck, Lübeck, Germany; 11 Free and Hanseatic City of Hamburg, Ministry of Justice and Consumer Protection, Hamburg, Germany; 12 Free and Hanseatic City of Hamburg, Ministry of Science, Research and Equality, Hamburg Cancer Registry, Hamburg, Germany; 13 Cancer Registry of Mecklenburg-Western Pomerania, Greifswald, Germany; 14 Hessian Office for Health and Care, Hessian Cancer Registry, Frankfurt/Main, Germany; 15 Clinical-Epidemiological Cancer Registry Brandenburg-Berlin, Cottbus, Germany; 16 Cancer Registry of North Rhine-Westphalia, Bochum, Germany; 17 Institute of Medical Informatics, Biometry, and Epidemiology, Medical Faculty, University of Duisburg-Essen, Essen, Germany; 18 German Childhood Cancer Registry, IMBEI, University Medicine at JGU Mainz, Mainz, Germany; 19 Department of Obstetrics and Gynecology, University Medical Center of Johannes Gutenberg University Mainz, Langenbeckstr. 1, 55131 Mainz, Germany

**Keywords:** Ovarian germ cell tumor, Ovarian sex cord stromal tumor, Cancer registry, Tumor characteristics, Therapy, Cancer, Oncology

## Abstract

Malignant ovarian germ cell tumors (MOGCT) and ovarian sex cord stromal tumors (SCST) are rare ovarian tumors. Since 2013, all German federal states are obliged to record data on diagnosis, pathology, therapy, and clinical course of tumor diseases. We gathered data on MOGCT and SCST from thirteen federal cancer registries and the German Childhood Cancer Registry. Each of the participating registries provided aggregated data on MOGCT and SCST with date of diagnosis between 01.01.2016 and 31.12.2021. 629 MOGCT and 872 SCST were included, age peaks at diagnosis for MOGCT and SCST were between 20 and 24 years and between 55 and 59 years, respectively. Of all MOGCT, 23%, 22%, 13% and 12% were malignant teratoma, dysgerminoma, neuroendocrine tumors and yolk sac tumors, respectively. The large majority (94%) of SCST represented malignant granulosa cell tumors. Most tumors were diagnosed in FIGO stage I (66% of MOGCT and 85% of SCST). Surgery was reported in 58% of patients with MOGCT and in 73% of patients with SCST. Chemotherapy was administered to 26% of MOGCT patients and 8% of SCST patients. This study highlights the great potential of cancer registries as high numbers of patients are recorded, however, data are highly dependent on the reporting institutions.

**Supplementary Information:**

The online version contains supplementary material available at 10.1038/s41598-025-32998-5.

## Introduction

 Malignant ovarian germ cell tumors (MOGCT) and ovarian sex cord stromal tumors (SCST) are rare ovarian neoplasms. They account for around 1.3% (MOGCT) and 1.6% (SCST) of malignant ovarian tumors in Europe^1^. MOGCT are a heterogeneous group of different tumors, the most common entities include dysgerminomas, immature teratomas and yolk sac tumors^2^. They mainly affect young women of childbearing age^3^. SCST mainly include adult and juvenile granulosa cell tumors and Sertoli-Leydig cell tumors and frequently affect middle-aged women^4^. The treatment of choice for MOGCT includes surgical staging including stage-appropriate surgical resection after surgical staging, and, for tumors of stages higher than FIGO IA, platinum-based chemotherapy (CHT) regimens including etoposide with addition of bleomycin or ifosfamide depending on the risk assessment^5^. Surgical treatment for SCST is also based on the staging analogous to ovarian cancer. The extent of the surgical resection depends on the extent of the tumor and, in case no hysterectomy is performed, includes a curettage of the corpus uteri to exclude simultaneous endometrial carcinoma. The advantage of adjuvant platinum-containing CHT after complete resection is discussed controversially but may be considered for stages > FIGO IC^5–8^. MOGCT and SCST are usually diagnosed in stage I (57% and 64%) and are associated with a good prognosis. Studies from the US National Center for Health Services show that MOGCT have a 5-year survival probability of 94% and 88% for SCST, respectively across all stages, the 5-year cause-specific survival for diagnosis in stage I is 99% (MOGCT) and 98% (SCST)^9^. However, recurrences are particularly common with SCST (10–64%) and can still occur late, on average 48–57 months after initial diagnosis^10^.

The Cancer Early Detection and Registry Act (Krebsfrüherkennungs- und -registergesetz; KFRG) (§ 65c, Social Code, Book V) obliges all federal states in Germany to record oncologic diseases in clinical cancer registries^11^. In addition, epidemiologic cancer registration of pediatric tumors has been in place on a national scale since the early 1980s^12^.

To study the epidemiologic characteristics of these groups of rare ovarian tumors and the standards of surgical and systemic therapy in Germany we gathered aggregated data of 13 federal cancer registries and the German Childhood Cancer Registry from 2016 – 2021.

## Methods

This study was based on pooled data from 13 German cancer registries i.e. clinical cancer registries of Rhineland-Palatinate, Schleswig-Holstein, Hamburg, Bremen, Mecklenburg-Western Pomerania, Brandenburg and Berlin, North Rhine-Westphalia, Lower Saxony, Hesse, Saxony-Anhalt, Saxony, Bavaria and Baden-Württemberg as well as the German Childhood Cancer Registry, representing more than 95% of the German population. Two cancer registries, Saarland and Thuringia did not deliver data for this study, data from Lower Saxony and Saxony-Anhalt were only available from 2018 to 2021 and from Hesse for the years 2016–2020. Each of the participating registries provided aggregated data on MOGCT and SCST for pooled analysis. Inclusion criteria were a primary diagnosis of ICD-10-GM C56 with one of the following ICD-O-3 histo-morphology codes (suppelement Table 1) (MOGCT: 8070/3, 8240/3, 8243/3, 8410/3, 9060/3–9065/3, 9070/3–9072/3, 9080/3- 9086/3, 9090/3, 9100/3, 9105/3 and SCST: 8600/3, 8620/3, 8630/3, 8631/3, 8634/3, 8640/3, 8650/3, 8670/3, 8810/3)^13,14^ and date of diagnosis between 01.01.2016 and 31.12.2021. Tumors with uncertain behavior were not included. For these patients aggregated data on age at diagnosis in five-year age groups, histo-morphology, FIGO 2014 staging classification^15^, grading, treatment, residual status, recurrences and vital status were provided. Numbers of 5 or less are not shown (< 6) to avoid de-anonymization. The pooled data was analyzed descriptively using R and RStudio (Version 3.6.3, 2020-02-29; Posit Software, PBC formerly RStudio, PBC, Boston, MA 02210, USA). Analyses were restricted to patients with available information for the variable of interest; we did not impute missing values. All methods were carried out in accordance with relevant guidelines and regulations, i.e. The Cancer Early Detection and Registry Act (Krebsfrüherkennungs- und -registergesetz; KFRG) (§ 65c, Social Code, Book V).

## Results

**Fig. 1:**
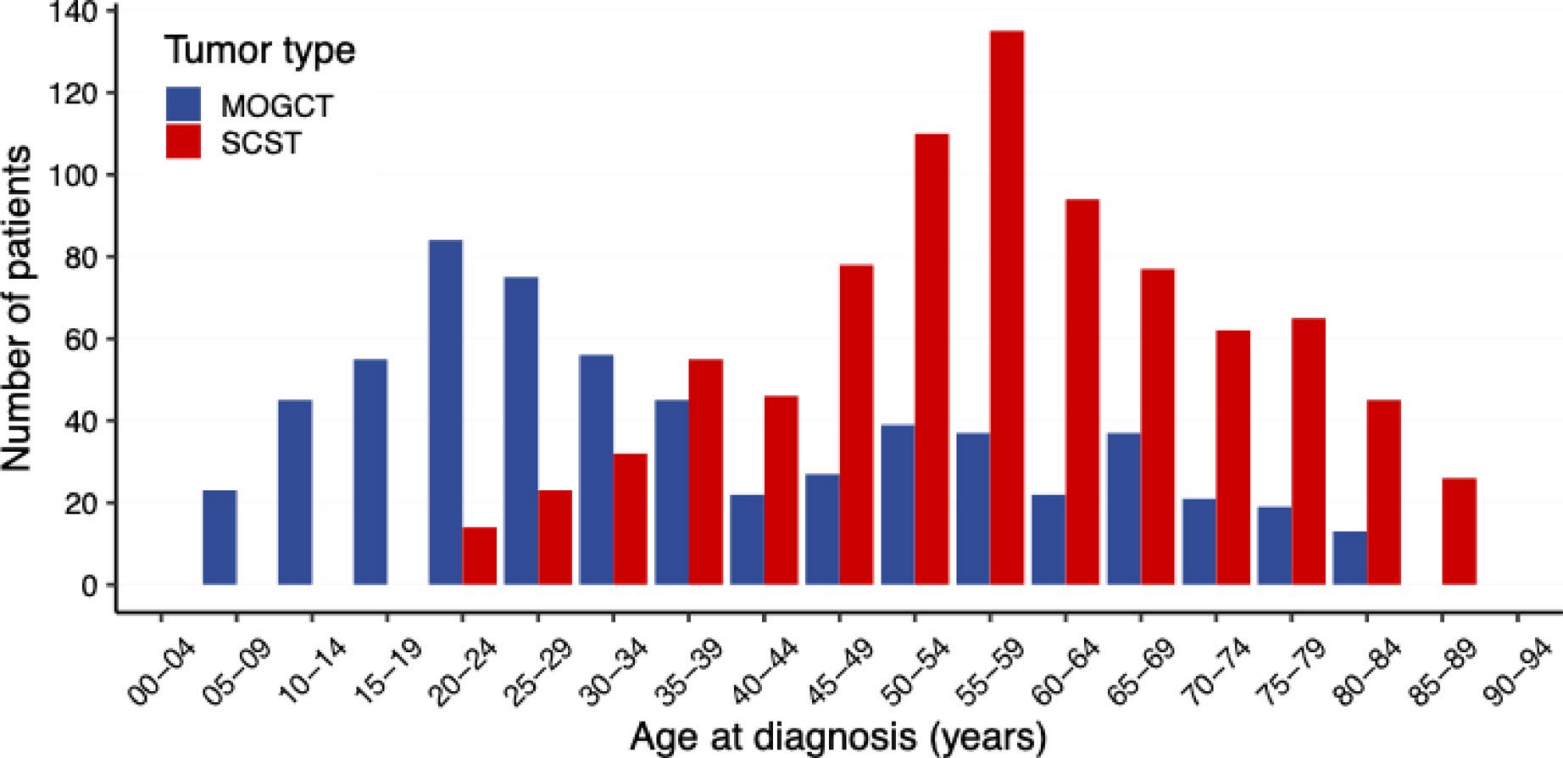
Age distribution of MOGCT and SCST in 5-year classes: incident cases of 2016–2021 (numbers < 6 not shown).

A total of 629 MOGCT and 872 SCST were included in the study. The age peaks of MOGCT and SCST were 20–24 years and 55–59 years, respectively (Fig. 1). With respect to histo-morphology about 23%, 22%, 13% and 12% of GCT were malignant teratoma (9080/3), dysgerminoma (9060/3), neuroendocrine tumors (8240/3) and yolk sac tumors (9071/3), respectively, more than 94% of SCST were malignant granulosa cell tumors (8620/3) and 2% Sertoli-Leydig cell tumors (8631/3) (Table 1).

Initial FIGO stage was available in 290 cases (46%) of MOGCT and 518 cases (59%) of SCST. Of these, 66% of MOGCT were diagnosed in FIGO stage I, 9% in FIGO II, 13% in FIGO III and 12% in FIGO IV. SCST were classified as FIGO I in 85%, FIGO II in 6%, FIGO III in 8% and FIGO IV in 2% of the patients. Tumor grading was available for 285 cases (45%) of MOGCT, and 160 cases (18%) of SCST. Medium grading was most common for MOGCT and low grading for SCST (Table 1).

**Table 1:** Tumor characteristics of MOGCT and SCST: incident cases of 2016–2021.

	MOGCT Total = 629		SCST Total = 872	
	*n*	%	*n*	%
*Histology*				
Malignant granulosa cell tumor of the ovary (8620/3)	n.a.	n.a.	821	94
Malignant teratoma (9080/3)	142	23	n.a.	n.a.
Dysgerminoma (9060/3)	136	22	n.a.	n.a.
Neuroendocrine tumor (8240/3)	84	13	n.a.	n.a.
Yolk sac tumor (9071/3)	74	12	n.a.	n.a.
Sertoli-Leydig cell tumor (8631/3)	n.a.	n.a.	21	2
Others	193	31	30	3
*FIGO*	*290/629*	*46*	*518/872*	*59*
I	191/290	66	438/518	85
II	27/290	9	30/518	6
III	37/290	13	39/518	8
IV	35/290	12	11/518	2
missing	339/629	54	354/872	41
*Grading*	*285/629*	*45*	*160/872*	*18*
low	111/285	39	86/160	54
medium	158/285	55	70/160	44
high	16/285	6	< 6/160	n.a.
missing	344/629	55	712/872	82

Percentage is calculated for all reported cases without missings.

Surgery was reported in 363 patients with MOGCT (58%) with laparotomy being more frequent than laparoscopy (42 vs. 26%). Diagnosis of SCST was followed by surgery in 633 cases (73%), with laparoscopy and laparotomy being similar in frequency (38 vs. 35%) (Table 2). For both types of tumors adnexectomy was most frequent (81% of MOGCT and 80% of SCST – if unilateral or bilateral adnexectomy was performed cannot be distinguished with the variables reported to the cancer registries) followed by omentectomy (40% and 53% respectively). Hysterectomy was more common in SCST than in MOGCT (51% and 23%). In rare cases (9% of MOGCT and 4% of SCST) ovarian cysts were resected. Lymphadenectomies (LNE) in general were rare, with paraaortal LNE being most frequent (8% of MOGCT and 6% of SCST) (Table 2).

**Table 2:** Reported treatment of MOGCT and SCST: incident cases of 2016–2021.

	MOGCT Total = 629		SCST Total = 872	
	*n*	%	*n*	%
*Surgery*	*363/629*	*58*	*633/872*	*73*
Laparoscopy	96/363	26	241/633	38
Laparotomy	153/363	42	223/633	35
missing data on type of operation	114/363	31	169/633	27
Adnexectomy*	293/363	81	507/633	80
Omentectomy*	147/363	40	334/633	53
Peritonectomy*	140/363	39	294/633	46
Hysterectomy*	84/363	23	325/633	51
ovarian cystectomy*	34/363	9	24/633	4
Paraaortal lymphadenectomy*	30/363	8	35/633	6
Other*	77/363	21	97/633	15
*Chemotherapy*	*165/629*	*26*	*68/872*	*8*
Carboplatin*	43/165	26	56/68	82
Cisplatin*	121/165	73	13/68	19
Paclitaxel*	34/165	21	47/68	69
Etoposide*	126/165	76	12/68	18
Bleomycin*	61/165	40	9/68	13
Ifosfamide*	32/165	19	0	
Other*	34/165	21	11/68	16
*Radiotherapy*	*14/629*	*2*	*< 6/872*	*< 1*

Percentage is calculated for all reported cases without missings; *multiple options per therapy possible.

Local R status was available for 76% of both, operated MOGCT and SCST. Local R0 was achieved in 92% (MOGCT) and 96% (SCST) of reported R statuses (Table 3).

CHT was reported for 26% and 8% of MOGCT and SCST, respectively (Table 2). For MOGCT etoposide was the main substance followed by cisplatin. A third substance was added less frequently (bleomycin 40% and ifosfamide 19%). For SCST carboplatin (56%) and cisplatin (13%) were the most frequent substances followed by paclitaxel (47%) (Table 2). Radiotherapy was rarely reported with 2% for MOGCT and less than 1% for SCST (Table 2).

For 8% of patients with MOGCT recurrences were reported and 6% died due to the cancer disease. In case of SCST for 5% of patients’ recurrences were reported and 2% died due to the cancer disease (Table 3).

**Table 3:** Success of treatment of MOGCT and SCST: incident cases 2016–2021 reported to German cancer registries followed until the 31 st of December 2021.

	MOGCT Total = 629		SCST Total = 872	
	*n*	%	*n*	%
*Surgery Total*	*363*	*100*	*633*	*100*
*Local R status*	*275/363*	*76*	*482/633*	*76*
0	252/363	92	462/633	96
1	9/363	3	14/633	3
2	14/363	5	6/633	1
x	88/363	24	151/633	24
*Follow-Up*				
Recurrences	49/629	8	45/872	5
Cancer-Caused Death	35/629	6	19/872	2

Percentage is calculated for all reported cases.

## Discussion

This study compiles data on rare ovarian tumors MOGCT and SCST. It includes data from 13 German cancer registries as well as the German Childhood Cancer Registry for the years 2016–2021, enabling the analysis of a high number of cases (*n* = 629 and *n* = 872) for those rare entities.

Data show the median ages at initial diagnosis, patients with MOGCT are considerably younger than patients with SCST (Fig. 1), a finding consistent with previous studies^3,4,16^. Although the initial FIGO stage is available for only half of the patients (46% of MOGCT and 59% of SCST) the available data indicate that both tumor types are predominantly diagnosed in FIGO stage I (66% and 85% respectively). This appears comparable to SEER data, although interpretation is limited due to the high rate of unknown staging information^9^.

Surgery was reported for 58% of MOGCT and 73% of SCST. This suggests incomplete reporting, as surgery is the treatment of choice for both tumor types^5,8^. Some missing surgical data may be explained by advanced, inoperable tumors, but this should only account for a minority of cases (approximately 25% MOGCT and 10% of SCST in our data).

Laparotomy was more frequent than laparoscopy in patients with MOGCT (42 vs. 26%), in patients with SCST both surgical approaches were similarly frequent (38 vs. 35%). While open surgery is generally preferred, minimally invasive staging may be appropriate in selected cases^8^. According to the German guideline on ovarian cancer, laparotomy is recommended for staging of SCST; however, laparoscopy is possible in early stages and in experienced centers^5^.

Adnexectomy was the most frequent procedure, however data of the cancer registries cannot distinguish between unilateral and bilateral adnexectomy, limiting insight into fertility sparing surgery rates.

Hysterectomy has been performed more often in patients with SCST than with MOGCT, which aligns with recommendations: hysterectomy is usually avoided in young MOGCT patients, whereas it may be omitted in patients with SCST only at stage IA^8^. However, cohort analysis showed that patients with SCST and unilateral adnexectomy had a worse 5-year survival than women with bilateral adnexectomy and hysterectomy^6^. The younger age of MOGCT patients likely explains the lower hysterectomy rates and the higher proportion of fertility-sparing procedures. In SCST, uterine curettage is obligatory, if hysterectomy is not performed, to rule out simultaneous endometrial carcinoma^5^; however, curettage data were not provided for this study.

Omentectomy and peritonectomy were performed in 40–50% of cases. Studies suggest higher recurrence rates in MOGCT patients without peritoneal biopsies, though without impact on overall survival^17^. LNE was rarely documented (MOGCT 8%, SCST 6%), consistent with guideline recommendations as LNE is not advised in the absence suspicious findings^5,8,18^.

R status was reported for 76% of operated patients, of whom 92% (MOGCT) and 96% (SCST) underwent complete (R0) resection.

CHT was administered to 26% of MOGCT patients and 8% of SCST patients. However, this is only comparable to a limited extent as there was a high number of patients with unknown stage. The CHT rate in MOGCT appears low given that the treatment is recommended for stages > FIGO IA. In SCST, there is no consensus regarding the benefit of CHT, although it may be considered for stages > FIGO IC^5^. This reflects both a potential heterogeneity and uncertainty in real-world practice and the impact of missing data. The substances most frequently used correspond to guideline recommendations: platinum-based combinations with etoposide (with or without bleomycin or ifosfamide) for MOGCT, and platinum combined mainly with paclitaxel for SCST. As expected, radiotherapy played no relevant role in treatment of MOGCT and SCST, although it is unclear whether reporting on radiotherapy was complete.

Recurrence rates were 8% (MOGCT) and 5% (SCST). These rates are slightly lower than reported in previous studies with 17.8%^19^ and 9%^20^ for MOGCT but substantially lower for SCST, where recurrence rates of 20%^6,21^ and up to 44% for granulosa cell tumors have been described^22^. The low SCST recurrence rate in our dataset is likely explained by the short observation period between 2016 and 2021. Patients may have experienced recurrence after the study period and thus remain unrecorded. Due to this limited follow-up window our study mostly reflects early events.

This study demonstrates the considerable potential of cancer registries to collect large cohorts, even for rare tumor types – numbers that are difficult to achieve even in large multicenter studies^23–29^. With these data, we can outline tumor characteristics and treatment patterns for MOGCT and SCST in Germany. However, despite the strength of the large cohort size, the substantial amount of missing information poses limitations and potential bias. Meanwhile cancer registries established several strategies to overcome this limitation^30^. Other limitations include the inability of pooled data to support more detailed descriptive analyses and the lack of central pathology review, despite the diagnostic complexity of these tumor types.

This is the first study to gather and publish the clinical data of nearly all German federal cancer registries on these entities into one analysis, enabling analysis of an unusually large cohort of these rare tumors. The major advantage of cancer registry data lies in its real-world nature. All inpatient and outpatient physicians are legally required to report diagnostic, treatment and follow-up information. However, the value of these data depends heavily on the quality and completeness of the submitted reports. Studies like ours may help motivate clinicians to improve reporting quality, thereby maximizing the utility of cancer registry data.

## Supplementary Information

Below is the link to the electronic supplementary material.

Supplementary Material 1

## Data Availability

All data generated or analysed by the cancer registries during this study are included in this published article. All data that support the findings of this study are listed in the references section.

## References

[1] Matz, M. et al. The histology of ovarian cancer: worldwide distribution and implications for international survival comparisons (CONCORD-2). *Gynecol. Oncol.* **144** (2), 405–413 (2017). 27931752 10.1016/j.ygyno.2016.10.019 PMC6195192

[2] Smith, H. O. et al. Incidence and survival rates for female malignant germ cell tumors. *Obstet. Gynecol.* **107** (5), 1075–1085 (2006). 16648414 10.1097/01.AOG.0000216004.22588.ce

[3] Zalel, Y. et al. Diagnosis and management of malignant germ cell ovarian tumors in young females. *Int. J. Gynaecol. Obstet.* **55** (1), 1–10 (1996). 8910077 10.1016/0020-7292(96)02719-1

[4] Schultz, K. A. et al. Ovarian sex Cord-Stromal tumors. *J. Oncol. Pract.* **12** (10), 940–946 (2016). 27858560 10.1200/JOP.2016.016261 PMC5063189

[5] Leitlinienprogramm Onkologie (Deutsche Krebsgesellschaft DK, AWMF). S3-Leitlinie Diagnostik, Therapie und Nachsorge maligner Ovarialtumoren. Langversion 6.1.01, 2025, AWMF-Registernummer: 032/035OL. https://www.leitlinienprogramm-onkologie.de/fileadmin/user_upload/LL_Ovarialkarzinom_Langversion_6.1.01.pdf , [Download 2025/12/17].

[6] Seagle, B. L., Ann, P., Butler, S. & Shahabi, S. Ovarian granulosa cell tumor: A National cancer database study. *Gynecol. Oncol.* **146** (2), 285–291 (2017). 28532858 10.1016/j.ygyno.2017.05.020

[7] Vergote, I. et al. Clinical research in ovarian cancer: consensus recommendations from the gynecologic cancer intergroup. *Lancet Oncol.* **23** (8), e374–e84 (2022). 35901833 10.1016/S1470-2045(22)00139-5 PMC9465953

[8] Ray-Coquard, I. et al. Non-epithelial ovarian cancer: ESMO clinical practice guidelines for diagnosis, treatment and follow-up. *Ann. Oncol.* **29** (Suppl 4), iv1–iv18 (2018). 29697741 10.1093/annonc/mdy001

[9] Torre, L. A. et al. Ovarian cancer statistics, 2018. *CA Cancer J. Clin.* **68** (4), 284–296 (2018). 29809280 10.3322/caac.21456 PMC6621554

[10] Levin, G., Zigron, R., Haj-Yahya, R., Matan, L. S. & Rottenstreich, A. Granulosa cell tumor of ovary: A systematic review of recent evidence. *Eur. J. Obstet. Gynecol. Reprod. Biol.* **225**, 57–61 (2018). 29665458 10.1016/j.ejogrb.2018.04.002

[11] Katalinic, A. et al. Population-Based clinical cancer registration in Germany. *Cancers (Basel)* ;2;**15**(15),3934. (2023). 10.3390/cancers15153934 PMC10416989 37568750

[12] Becker, C. et al. Early deaths from childhood cancer in Germany 1980–2016. *Cancer Epidemiol.* **65**, 101669 (2020). 31955037 10.1016/j.canep.2020.101669

[13] Carcangiu, M. L., Kurman, R. J., Carcangiu, M. L., Herrington, C. S. & WHO Classification of Tumours of Female Reproductive Organs. Lyon: International Agency for Research on Cancer; (2014). Available from: http://VH7QX3XE2P.search.serialssolutions.com/?V=1.0&L=VH7QX3XE2P&S=JCs&C=TC0001705053&T=marc&tab=BOOKS

[14] (BfArM) BfAuM. ICD-O-3 Zweite Revision: BfArM. (2023). Available from: https://www.dimdi.de/dynamic/de/klassifikationen/icd/icd-o-3/icd03rev2html/chapter-m.htm

[15] Wittekind, C. *TNM: Klassifikation Maligner Tumoren* 336 (Wiley, 2017).

[16] Saani, I. et al. Clinical challenges in the management of malignant ovarian germ cell tumours. *Int. J. Environ. Res. Public. Health* ;**20**(12), 6089. (2023). 10.3390/ijerph20126089 PMC10298722 37372675

[17] Mangili, G. et al. The role of staging and adjuvant chemotherapy in stage I malignant ovarian germ cell tumors (MOGTs): the MITO-9 study. *Ann. Oncol.* **28** (2), 333–338 (2017). 27803008 10.1093/annonc/mdw563

[18] Nasioudis, D., Kanninen, T. T., Holcomb, K., Sisti, G. & Witkin, S. S. Prevalence of lymph node metastasis and prognostic significance of lymphadenectomy in apparent early-stage malignant ovarian sex cord-stromal tumors. *Gynecol. Oncol.* **145** (2), 243–247 (2017). 28292524 10.1016/j.ygyno.2017.03.005

[19] Mangili, G. et al. Outcome and risk factors for recurrence in malignant ovarian germ cell tumors: a MITO-9 retrospective study. *Int. J. Gynecol. Cancer*. **21** (8), 1414–1421 (2011). 21795985 10.1097/IGC.0b013e3182236582

[20] Saeed Usmani, A., Yasin, I., Asif, R. B., Kahlid, N. & Syed, A. Incidence and survival rates for female malignant germ cell tumors: an institutional review. *Cureus* **14** (4), e24497 (2022). 35651446 10.7759/cureus.24497 PMC9135046

[21] Li, J. et al. Progress in the management of ovarian granulosa cell tumor: A review. *Acta Obstet. Gynecol. Scand.* **100** (10), 1771–1778 (2021). 34027996 10.1111/aogs.14189

[22] Klar, M. et al. Treatment and survival of patients with malignant ovarian sex cord-stromal cell tumours: an analysis of the arbeitsgemeinschaft fur Gynakologische onkologie (AGO) study group CORSETT database. *J. Surg. Oncol.* **128** (1), 111–118 (2023). 36975108 10.1002/jso.27248

[23] Minig, L. A. O. et al. Oncological outcomes among young women with non-epithelial ovarian cancer: the YOC-Care study (Young ovarian Cancer - Care). *Int. J. Gynecol. Cancer*. **33** (6), 915–921 (2023). 36796862 10.1136/ijgc-2022-004162

[24] Lenck, C. et al. The French National network dedicated to rare gynecological cancers diagnosis and management could improve the quality of surgery in daily practice of granulosa cell tumors. A TMRG and GINECO group study. *Gynecol. Oncol.* **157** (1), 78–84 (2020). 32131977 10.1016/j.ygyno.2020.02.012

[25] Sun, H. D. et al. A long-term follow-up study of 176 cases with adult-type ovarian granulosa cell tumors. *Gynecol. Oncol.* **124** (2), 244–249 (2012). 22019525 10.1016/j.ygyno.2011.10.015

[26] Sehouli, J. et al. Granulosa cell tumor of the ovary: 10 years Follow-up data of 65 patients. *Anticancer Res.* **24**, 1223–1230 (2004). 15154651

[27] Wilson, M. K. et al. Stage I granulosa cell tumours: A management conundrum? Results of long-term follow up. *Gynecol. Oncol.* **138** (2), 285–291 (2015). 26003143 10.1016/j.ygyno.2015.05.011

[28] Pectasides, D. et al. Adult granulosa cell tumors of the ovary: A clinicopathological study of 34 patients by the Hellenic cooperative oncology group (HeCOG). *Anticancer Res.* **28**, 1421–1428 (2008). 18505090

[29] Hasenburg, A. et al. The effect of fertility-sparing surgery on sexuality and global quality of life in women with malignant ovarian germ cell and sex cord stromal tumors: an analysis of the CORSETT database of the AGO study group. *Arch. Gynecol. Obstet.* **304** (6), 1541–1549 (2021). 34287678 10.1007/s00404-021-06019-5 PMC8553700

[30] Plachky, P. et al. *Benefits of the Introduction of a Monitoring Program and Field Crew Service in Cancer Registration in Rhineland-Palatinate* Vol. 36 (Oncology Research and Treatment, 2024).

