# Supplementary material for: Epidemiology and treatment of malignant ovarian germ cell and sex cord stromal tumors in germany: a population-based cancer registry study from 2016 – 2021
Source: Sci Rep. 2025 Dec 18;15:44145. doi: 10.1038/s41598-025-32998-5 (PMC12717222; doi:10.1038/s41598-025-32998-5)
Supplement: Supplementary file 1 — Supplementary Material 1 [file 41598_2025_32998_MOESM1_ESM.docx]

Supplement

Table 1: Listing of all ICD-O-3 morphology codes included for analysis.

| 8070/3 | MOGCT | Squamous cell carcinoma, NOS |
| --- | --- | --- |
| 8240/3 | MOGCT | Carcinoid tumor, NOS |
| 8243/3 | MOGCT | Goblet cell carcinoid |
| 8600/3 | SCST | Thecoma, malignant (C56.9) |
| 8620/3 | SCST | Granulosa cell tumor, malignant (C56.9) |
| 8630/3 | SCST | Androblastoma, malignant |
| 8631/3 | SCST | Sertoli-Leydig cell tumor, poorly differentiated |
| 8634/3 | SCST | Sertoli-Leydig cell tumor, poorly differentiated, with heterologous elements |
| 8640/3 | SCST | Sertoli cell carcinoma (C62._) |
| 8650/3 | SCST | Leydig cell tumor, malignant (C62._) |
| 8670/3 | SCST | Steroid cell tumor, malignant |
| 8810/3 | SCST | Fibrosarcoma, NOS |
| 9060/3 | MOGCT | Dysgerminoma |
| 9064/3 | MOGCT | Germinoma |
| 9070/3 | MOGCT | Embryonal carcinoma, NOS |
| 9071/3 | MOGCT | Yolk sac tumor |
| 9080/3 | MOGCT | Teratoma, malignant, NOS |
| 9081/3 | MOGCT | Teratocarcinoma |
| 9082/3 | MOGCT | Malignant teratoma, undifferentiated |
| 9083/3 | MOGCT | Malignant teratoma, intermediate |
| 9084/3 | MOGCT | Teratoma with malignant transformation |
| 9085/3 | MOGCT | Mixed germ cell tumor |
| 9090/3 | MOGCT | Struma ovarii, malignant (C56.9) |
| 9100/3 | MOGCT | Choriocarcinoma, NOS |

(MOGCT= malignant ovarian germ cell tumor; SCST= sex cord stromal tumor; NOS= not otherwise specified)
